# Impact of the COVID-19 pandemic on the Emergency Department of a tertiary children’s hospital

**DOI:** 10.1186/s13052-021-00976-y

**Published:** 2021-01-29

**Authors:** Umberto Raucci, Anna Maria Musolino, Domenico Di Lallo, Simone Piga, Maria Antonietta Barbieri, Mara Pisani, Francesco Paolo Rossi, Antonino Reale, Marta Luisa Ciofi degli Atti, Alberto Villani, Massimiliano Raponi

**Affiliations:** 1 Pediatric Emergency Department, Bambino Gesù Children’s Hospital, IRCCS, Rome, Italy; 2 Medical Direction, Bambino Gesù Children’s Hospital, IRCCS, Rome, Italy; 3 Clinical Pathways and Epidemiology Unit, Medical Direction, Bambino Gesù Children’s Hospital, IRCCS, Rome, Italy; 4 Pediatric Emergency Department, Bambino Gesù Children’s Hospital, IRCCS, Palidoro, Rome, Italy

**Keywords:** COVID-19, Emergency department, Diagnostic delay, Preparedness, Children

## Abstract

**Background:**

Italy was the first country in Europe affected by COVID-19: the emergency started on February 20, 2020, culminating with national lockdown on March 11, which terminated on May 4, 2020. We describe how the pandemic affected Emergency Department (ED) accesses in a tertiary children’s hospital, composed by two different pediatric centers, one located in Rome’s city center and the second, Palidoro (regional COVID-19 center), in its surrounding metropolitan area, both in the Lazio region, analyzing the profile of admitted patients during the pandemic period in terms of their general characteristics (at presentation in the ED’s) and urgent hospitalizations compared to prepandemic period.

**Methods:**

The study compare the period between the 21st of February and the 30th of April 2020, covering the three phases of the national responses (this period will be referred to as the pandemic period) with the same period of 2019 (prepandemic period). The study analyzes the number of ED visits and urgent hospitalizations and their distribution according to selected characteristics.

**Results:**

The reduction of ED visits was 56 and 62%, respectively in Rome and Palidoro centers. The higher relative decline was encountered for Diseases of Respiratory System, and for Diseases of the Nervous System and Sense Organs. A doubling of the relative frequency of hospitalizations was observed, going from 14.2 to 24.4% in Rome and from 6.4 to 10.3% in Palidoro. In terms of absolute daily numbers the decrease of urgent hospitalizations was less sharp than ED visits. For pathologies such as peritonitis, tumors or other possible life-treathening conditions we did not observe a significative increase due to delayed access.

**Conclusions:**

In the pandemic period there was a general reduction in the number of children referred to ED, such reduction was greater in low-acuity levels. The reduction for respiratory tract infections and other communicable diseases during school closure and the national lockdown must make us reflect on the possible impact that these conditions may have on the health system, in particular the ED, at the reopening of schools. The major problem remains the fear for possible diagnostic delays in life-threatening or crippling diseases; our study doesn’t demonstrate an increase in number or significant delay in some serious conditions such as tumors, peritonitis, diabetic ketoacidosis, ileo-colic intussusception and testis/ovary torsion. A continuous, deep re-organizational process step by step of the ED is nececessary in the present and upcoming pandemic situation.

## Introduction

Since the outbreak of Coronavirus Disease 2019 (COVID-19) in the city of Wuhan in China, in the early December 2019 the COVID-19, the pandemic has taken the world by storm and ravaged almost every country in the world. In fact on January 30th 2020, the World Health Organization (WHO) announced that the COVID-19 outbreak was a public health emergency of international concern and on March 11th the WHO described the COVID-19 outbreak as a pandemic [1]. Italy was the first affected country in Europe, with the first person-to-person transmission diagnosed on February 20th 2020. National response actions to contain the pandemic upgraded from strict social distancing measures in 11 municipalities in Northern Italy, on February 23rd 2020, to national social distancing and school closures on March 4th 2020 and culminated with the national lockdown on March 11th 2020, terminated on the May 4th 2020 [2].

Children are affected less frequently and with more benign disease compared to adults [3]; they are in fact accounted for about 2.2% of the diagnosed cases in China [4], 2.4% in Australia [5], 2.3% in the United States [6, 7] and 3.3% in Norway [8]. In Italy, overall, the diagnosed cases in the “<18 years old” age group are 2.0% of the total; among them, 12.5% ​​are imputable to children younger than 1 year old, 18.1% to children between 2 and 6 years old and 69.4% to children between 7 and 17 years old [9]. In the United States, the percentage of children with COVID-19 needing hospitalization was between 1.6 and 2.5% [6].

Healthcare facility preparedness is a key component of the response to the COVID-19 pandemic [10] and it is crucial to ensure appropriate space, supplies and personnel, prioritizing care, activating triage procedures and training staff on infection prevention, control and clinical management for COVID-19. A survey, conducted through pediatric emergency medicine research networks (REPEM) and United Kingdom and Ireland (PERUKI), highlithed differences and gaps in preparedness and response to the COVID-19 pandemic, a lack in early documented contingency plan, provision of simulation training, appropriate use of Personal Protective Equipment (PPE) and appropriate isolation facilities [11].

Emergency departments (ED), also in pediatric hospitals, are on the frontlines, serving an essential and primary function in identifying patients with suspected COVID-19, isolating them early whilst providing urgent medical care. A re-organizational process of the ED was undertaken since entering the hospital and therefore from triage of the child and his parents to final disposition because the use of specific strategy ways is known to offer a buffer solution in many disaster situations. Hospital and in particular ED must be constantly resilient and prepared to these new emergencies in terms of equipment, medical and nurses staff, peculiar bed capacity in short time, availability of intensive and sub-intensive beds, and flexibility [12, 13].

During the national lockdown of Italy for COVID-19 a substantial decrease in paediatric ED visits was observed and similarly, family paediatricians widely reported a considerable reduction in clinic visits [14]. The possibility of postponing necessary urgent care for conditions with possible serious consequences has been advocated in Italy [14, 15] and in other countries [16–18] and this could have been a contributing factor of death in some children [19].

In this article, we describe how the pandemic affected ED access in a tertiary care children’s hospital in central Italy, comparing the profile of admitted patients in the initial COVID period with the same period of the previous year.

### Study setting

The study was conducted in the Bambino Gesù Children’s Hospital [Ospedale Pediatrico Bambino Gesù (OPBG)], an academic tertiary care children’s hospital, with 607 inpatient beds, composed by two different pediatric centers, one located in Rome’s city center and the second, Palidoro, in its surrounding metropolitan area, both in the Lazio region. The EDs provide free urgent medical care to pediatric population as the only children’s hospital in the Lazio region: during 2019 there were 89,558 ED visits and 11,519 urgent inpatient admissions.

On January 24th 2020, OPBG was designated as Lazio’s regional hub for children with COVID-19 [20]. The hospital promptly developed a rapid assessment and change in triage procedures, in order to enable emergency pediatricians to initially evaluate any children presenting to the ED. ED flows were organized to separate children with fever and respiratory symptoms from other patients and Infection Prevention and Control measures were enforced. At the regional level, individuals with fever and respiratory symptoms were discouraged from directly going to ED, and encouraged to call their general practitioners or the regional health emergency center, and follow the instructions given based on clinical history, signs and symptoms. If COVID-19 was suspected and the patient’s conditions were suggestive of clinical deterioration, the regional ambulance service was in charge of transporting the patient to the hospital. Starting from February 23rd 2020, all patients and their parents were actively screened when entering the hospital using a structured questionnaire checking for fever, respiratory symptoms and possible contacts with COVID-19 cases. Only one parent or relative per patient was allowed to enter the hospital and nonurgent outpatient visits were suspended.

Suspected COVID-19 cases, transported by regional pre-hospital emergency service, were admitted directly to regional COVID center of OPBG in Palidoro.

## Material and methods

The study compared the period between the 21st February and the 30st April 2020, covering the three phases of the national responses (the first Italian case, the start and the subsequent lockdown or stay-at-home phase), which will be referred to as *pandemic period,* with the same period of 2019 *prepandemic period*.

We collected data from the OPBG Healthcare Emergency Information System (HEIS) and the OPBG Hospital Information System (HIS). The HEIS collects information related to all visits to ED, such as patient demographics, admission information including triage score, visit and discharge dates and hours, ICD-9-CM diagnosis at discharge, status at discharge (i.e., dead, hospitalized, or discharged at home). The hospital HIS is an integrated information system designed to collect clinical and administrative information regarding hospital admissions for each patient discharged from the hospital.

We analyzed, on both centers and periods, the number of ED visits and urgent hospitalizations and their distribution according to selected characteristics: age (< 1, 1–5, 6–11, > 12 years), sex, mode of arrival (Autonomous, Ambulance, Helicopter), priority of consultation (High/intermediate and Low/Non urgent) derived from the triage system, selected ICD-9-CM chapters (Neoplasms; Diseases of the blood and blood-forming organs and certain disorders involving the immune mechanism; Mental disorders; Diseases of the Nervous System and Sense Organs; Circulatory System; Diseases of the Respiratory System; Digestive System; Congenital Anomalies; Injury and Poisoning), urgent hospitalization with lenght of stay, hospitalization area (Medical, Surgical, Oncoematologic, Psichiatric, Critical), the ICD-9-CM chapters at discharge, complicated appendicitis (ICD-9 codes 540.0, 540.1, 567.9) as a proxy example of possible delay to ED, day of the week and the time (hours) of access to the ED. In addition, we also considered the following conditions: diabetic ketoacidosis, ileo-colic intussusception and testis/ ovary torsion.

### Statistical analysis

Categorical data was summarized as counts and percentages, while continuous data was analyzed as means and SDs. To take into account the different duration of the two periods (i.e. 69 days the first and 70 days the second), the numbers of visits and urgent hospitalizations were analyzed in terms of daily mean numbers. To determine statistical differences when comparing the two periods, the χ2 test for categorical variables and independent samples t-test for continuous variables were used. A 2-sided *p*-values of less than 0.05 defined statistical significance. All statistical analyses were performed using STATA, Statistical Software: Release 13 (StataCorp LP, College Station, TX).

## Results

**Fig. 1:**
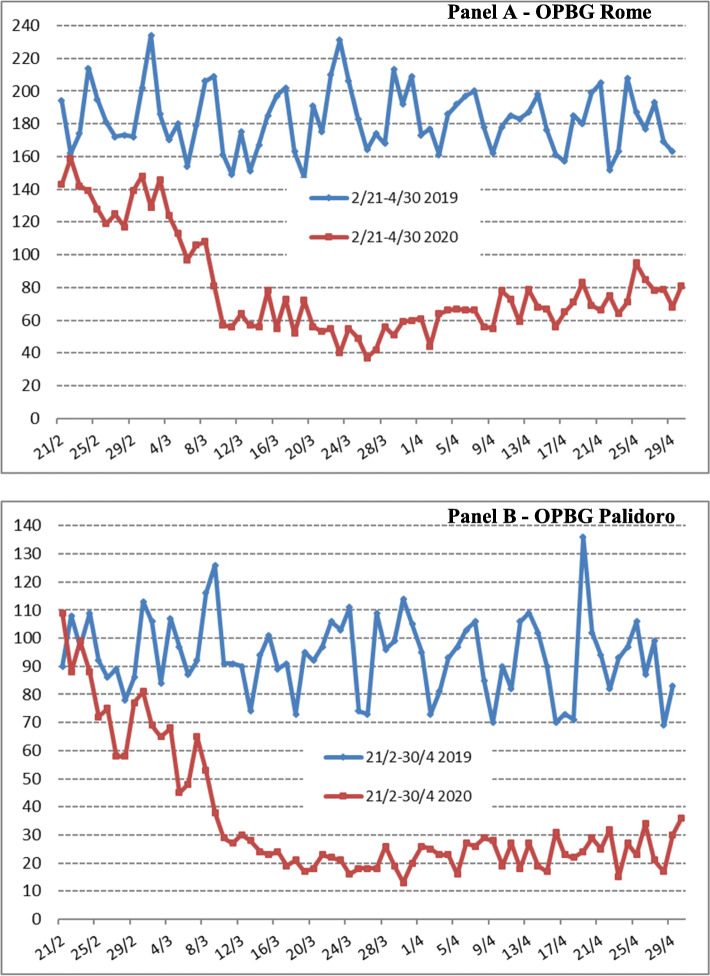
Total daily ED visits by center over the two study periods

Starting from February 21st 2020, ED visits declined sharply until April 30th 2020, compared to the same period in 2019 in both our ED centre (Fig. 1). In fact, the reduction of ED visits between pandemic and prepandemic period in OPBG Rome and Palidoro center was 56 and 62% respectively (Table 1). For both centers there was a significant statistical decrease in daily mean numbers of ED visits in all age groups and sexes. Due to its function of paediatric COVID regional hospital, a significant increase (+ 129%) of ambulance transport to ED was observed in the OPBG Palidoro.

**Table 1:** Emergency Department visits by selected characteristics. Comparison between prepandemic and pandemic periods in the two centers of a tertiary children’s hospital

Emergency Department Visits	OPBG Rome							OPBG Palidoro						
	*Prepandemic Period*^*a*^		*Pandemic period*^*a*^		*p-value*^*#*^	*p-value*^*&*^	*% Change*	*Prepandemic Period*^*a*^		*Pandemic period*^*a*^		*p-value*^*#*^	*p-value*^*&*^	*% Change*
	N.	%	N.	%				N.	%	N.	%			
**Total**	12,602		5572			< 0.001	−56	6475		3469			< 0.001	−62
**Age, Years**					< 0.001							0.103		
< 1	2067	16.4	930	16.7		< 0.001	−55	657	10.1	257	10.4		< 0.001	− 61
1–5	5436	43.1	2242	40.2		< 0.001	−59	2875	44.4	1039	42.1		< 0.001	−64
6–11	3188	25.3	1396	25.1		< 0.001	−56	1925	29.7	738	29.9		< 0.001	−62
≥ 12	1911	15.2	995	17.9		< 0.001	−48	1017	15.7	434	17.6		< 0.001	−57
**Sex**					0.103							0.584		
Male	7017	55.7	3025	54.3		< 0.001	−57	3559	55.0	1373	55.6		< 0.001	−61
Female	5585	44.3	2538	45.5		< 0.001	−55	2916	45.0	1096	44.4		< 0.001	−62
**Mode of Arrival**					< 0.001							< 0.001		
Autonomous	11,656	92.5	4964	89.1		< 0.001	−57	6382	98.6	2256	91.4		< 0.001	−65
Ambulance	935	7.4	602	10.8		< 0.001	−36	93	1.4	213	8.6		< 0.001	+ 129
Helicopter	11	0.1	6	0.1		< 0.001	−45	–	–	–			–	
**Priority Consultation**					< 0.001							< 0.001		
High/Intermediate	2973	23.6	2017	36.2		< 0.001	−32	580	9.0	654	26.5		0.247	+ 13
Low/Non Urgent	9629	76.4	3555	63.8		< 0.001	−63	5895	91.0	1815	73.5		< 0.001	−69
**Selected ICD-9 CM Chapters**					< 0.001							< 0.001		
Neoplasms	55	0.4	55	1.0		0.942	0	2	0.0	5	0.2		0.256	+ 150
Blood and Blood Forming Organs	105	0.8	56	1.0		< 0.01	−47	69	1.1	14	0.6		< 0.001	−80
Mental Disorders	195	1.5	143	2.6		< 0.01	−27	21	0.3	16	0.6		0.404	−24
Nervous System and Sense Organs	776	6.2	225	4.0		< 0.001	−71	481	7.4	113	4.6		< 0.001	−77
Circulatory System	71	0.6	52	0,9		0.05	- 27	23	0,4	14	0,6		0.982	−39
Respiratory System	3013	23.9	1118	20.1		< 0.001	−63	1638	25.3	650	26.3		< 0.001	−60
Digestive System	475	3.8	273	4.9		< 0.001	−43	173	2.7	79	3.2		< 0.001	−54
Congenital Anomalies	237	1.9	130	2.3		< 0.001	−45	57	0.9	20	0.8		< 0.001	−65
Injury and Poisoning	3097	24.6	1643	29.5		< 0.001	−47	1651	25.5	638	25.8		< 0.001	−61
**Place/Type of Accident**					< 0.001							< 0.001		
Home	527	4.2	811	14.6		< 0.001	+ 54	358	5.5	305	5.5		< 0.05	−15
School	267	2.1	27	0.5		< 0.001	−90	258	4.0	32	1.3		< 0.001	−88
Sport	298	2.4	62	1.1		< 0.001	− 79	251	3.9	52	2.1		< 0.001	−79
Road Traffic	52	0.4	16	0.3		< 0.01	−69	21	0.3	1	0.0		< 0.001	−95
Other Places	783	6.2	258	4.6		< 0.001	−67	493	7.6	127	5.1		< 0.001	−74

^a^ Prepandemic corresponds to February 21–April 30, 2019 and Pandemic to February 21–April 30, 2020; # chi-squared & t-test comparing differences in mean daily numbers of ED visits between the two periods

According to the priority code, divided into two types of consultation priorities (high/intermediate and low/non-urgent conditions), a reduction in both groups was observed in PBG Rome, more evident in postponable-non urgent group (− 32% vs 63% of reduction), whereas at OPBG Palidoro the high/intermediate priority of consultations slightly increased (see Table 1 for detail).

In Table 1 the results of the main diagnosis at ED grouped in some selected ICD-9--CM chapters are summarized: in both centers the higher relative decline in the mean daily number of ED visits was most evident for Diseases of Respiratory System (− 63% in Rome and − 60% in Palidoro), mainly in children aged less than 12 years old (data not shown) and for Diseases of the Nervous System and Sense Organs (− 71% in Rome and − 77% in Palidoro). As expected, following the national lockdown on March 11th 2020, a statistical significant decrease of ED visits by place or type of accidents was observed for all groups except for injury in the domestic environment (+ 54% in OPBG Rome). No significant differences between the two periods for both the centers were observed in the relative distribution of ED visits by day of the week and the hour of the day (data not shown).

A doubling of the relative frequency of hospitalizations was observed, going from 14.2 to 24.4% in Rome center and from 6.4 to 10.3% in Palidoro center. In our Palidoro COVID center were hospitalized 42 children with SARS-CoV-2 infections and only one with important neurological comorbidity required O2 supplementation with low flow oxygen. The causes of admission were respiratory symptoms (in five interstitioal pneumonia at ED access), or gastrointestinal disorders, or neurological disorders such as febrile convulsions, or subject with important high risk comorbidity.

**Fig. 2:**
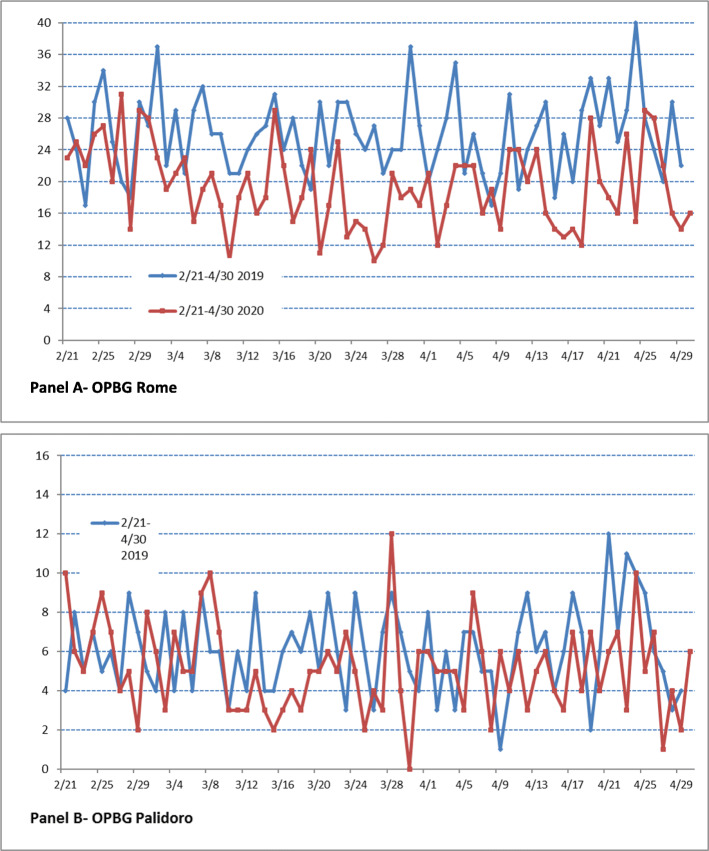
Total daily urgent hospital admissions by center over the two study periods

In terms of absolute daily numbers the decrease of urgent hospitalizations was less sharp than ED visits, from 1788 inpatient stays during prepandemic periods to 1360 during the pandemic period (24% reduction), and from 418 inpatient stays during prepandemic periods to 359 during the pandemic period (15% reduction), respectively in Rome and Palidoro centre (Table 2 and Fig. 2). Tacking into account clinical hospitalization area we observed a significant statistical decrease in the daily mean numbers for medical, surgical and critical area in Rome centre and only for medical hospitalization in Palidoro centre. The discharge principal diagnosis grouped in the same selected ICD-9-CM chapters choiced for ED visits are summarized in Table 2; in the Rome ED the relative decrease reached a statistical significance for all ICD-9-CM chapters analyzed (except for that of Congenital Anomalies where there was a slightly increase), while in the Palidoro center the statistically significant decrease was observed only for Disease of the Nervous System and Organ Sense. A reduction of ED admission for headache were present (268 cases in the prepandemic period to 95 cases in the pandemic with an overall change of – 64.6%). We must note that home accidents accounted for 24% of the urgent hospitalizations in the Injury and Poisoning chapter. As proxy for probable delayed access to the emergency room we extracted from the Hospital Information System database the surgical discharges with a severe complicated appendicitis (e.g. appendicitis with peritonitis or unspecified peritonitis). Overall, complicated forms of appendicitis (peritonitis) did not increase either in absolute terms (25 in prepandemic vs 17 in pandemic period) or as a percentage of the total appendicitis (48% vs 28%); in addition, symptom onset averaged 39.8 h (range 12–120 h) in the prepandemic period versus 36 h (range 6–120 h) in the pandemic.

**Table 2:** Emergency Department hospital admissions by selected characteristics. Comparison between prepandemic and pandemic periods in the two centres of a tertiary children’s hospital

Emergency Department Hospital Admissions	OPBG Rome							OPBG Palidoro						
	*Prepandemic Period*^*a*^		*Pandemic period*^*a*^		*p-value*^*#*^	*p-value*^*&*^	*% Change*	*Prepandemic Period*^*a*^		*Pandemic period*^*a*^		*p-value*^*#*^	*p-value*^*&*^	*% Change*
	N.	%	N.	%				N.	%	N.	%			
**Total**	1788	14.2^*b*^	1359	24.4^*b*^		< 0.001	−47	417	6.4^*b*^	358	10.3^*b*^		< 0.001	−14
**Hospitalization Area**					0.002							0.024		
Medical	701	39.2	571	42.0		< 0.001	−19	192	46.0	130	36.3		< 0.001	−32
Surgical	502	28.1	316	23.3		< 0.001	−37	209	50.1	209	58.4		0.965	0
Oncoematologic	27	1.5	40	2.9		0.250	+ 48	–	–	–	–		–	–
Psychiatric	60	3.4	53	3.9		0.441	−12	–	–	–	–		–	–
Critical	498	27.9	379	27.9		< 0.001	−24	16	3.8	18	5.0		0.968	+ 13
**Selected ICD-9 CM Chapter**					0.046							0.120		
Neoplasms	77	4.3	53	3.9		< 0.05	−31	–	–	–	–		–	–
Blood and Blood Forming Organs	69	3.9	45	3.3		< 0.05	−35	13	3.1	12	0.6		0.974	−8
Mental Disorders	82	4.6	72	5.3		0.359	−12	5	1.2	2	0.6		0.457	−60
Nervous System and Sense Organs	107	6.0	84	6.2		< 0.01	−21	41	9.8	12	4.6		< 0.05	−71
Circularity System	41	2.3	37	2.7		0.589	−10	8	1.9	13	3.6		0.254	+ 63
Respiratory System	317	17.7	165	12.1		< 0.001	−48	112	6.9	86	26.3		0.363	−23
Digestive System	144	8.1	129	9.5		0.383	−10	40	9.6	23	6.4		0.05	−43
Congenital Anomalies	43	2.4	51	3.8		< 0.713	+ 19	5	1.2	3	0.8		0.986	−40
Injury and Poisoning	253	14.1	213	15.7		< 0.05	−16	39	9.4	27	7.5		0.659	−31

^a^ Prepandemic corresponds to February 21–April 30, 2019 and Pandemic to February 21–April 30, 2020

^b^ percentage of total Hospital Admissions

# chi-squared test; & t-test comparing differences in mean daily numbers of ED Hospital admissions between the two periods

Moreover, we did not observe an increase of complicated diabetes (e.g. ketoacidosis) in absolute number, from 11 cases in the prepandemic period, 4 cases in patients with known diabetes mellitus type 1 and 7 cases in new onset diabetes, to 3 cases,all new onset diabetes, in the pandemic. Symptom onset of new onset diabetes averaged 23.7 days (range 1–90 days) in the prepandemic period versus 13 days (range 2–30 days) in the pandemic. All patients with ketoacidosis with yet known diabetes mellitus type 1 (all in prepandemic period) accessed in ED after 6–12 h from the onset of symptoms.

Other causes of severe abdominal pathology are considered, such as ileo-colic intussuaception and torsion of testis/ovary. We have not noticed a mild decrease of ileo-colic intussusception in absolute terms (9 patients in prepandemic period versus 6 in the pandemic) and torsion of testis/ovary (11 patients in prepandemic period versus 8 in the pandemic). In addition, symptom onset averaged 24.3 h (range 3–72) in ileo-colic intussusceptions and 30.6 h (range 1–96 h) in torsion of testis/ovary in the prepandemic period versus 16.6 h (range 4–48 h) in ileo-colic intussusception and 15.9 (range 2–48 h) in torsion of testis/ovary in the pandemic.

## Discussion

In the weeks following the declared state of national emergency for COVID-19 our data shows a significant decrease in presentations to our ED across nearly all categories, indicating that the measures adopted to limit the pandemic had a profound impact on the use of pediatric ED, with a 56% (Rome OPBG ED) and 62% (Palidoro OPBG ED) reduction in ED visits and a reduction in urgent hospitalizations in absolute number but not in percentage, compared to the same timespan of the previous year. These data are in agreement with the literature emerged in COVID-period in other Italian pediatric EDs (ranging from 72 to 88% of reduction) [14, 15, 21–23], but in our hospital the reduction percentage was lower in both centers. This difference could be due to various factors such as the diversification of our 2 centers (Rome and Palidoro-dedicated COVID regional center), the relationship with family paediatrician and hospital network, the implementation of tools such as telemedicine, and the dedicated teleconsultation for doctors and family members as well as an early documented contingency plan, continuously implemented by the hospital health management; furthermore, the diversity in relation to the regional distribution of the incidence rates of COVID-9 (low, medium, high) should be kept in mind with Lazio in the condition of a lower incidence rate and having mortality from coronavirus five times lower than the national average.

Often time people use services requiring emergency and urgent care though it is judged clinically unnecessary, and in pediatrics this phenomenon is even more marked. A recent review identified six reasons for attendance at directly ED services: the need for risk minimization, speed medical consult, low treatment-seeking burden compliance, consumer satisfaction, and frustration, where patients had attempted and failed to obtain a general practitioner appointment in the desired timeframe [24]. So, the overflow of patients to ED and the relative crowding may cause long waits, patient and parent anger, stress for the emergency physician, with possible subsequent drop in the care quality.

Nevertheless, the COVID-19 pandemic has challenged in large scale healthcare systems and people’s daily life. National media campaigns reinforced the policy message asking families to stay home to avoid the spreading of the infection. Previous preliminary reports seem to indicate a delayed access or provision of care because parents, or their guardians, brought their children to hospital later than they had in the past, with possible increase in avoidable morbidity and mortality [14, 15]. In order to optimize the healthcare response, hospitals have been reorganized, elective activities drastically reduced and COVID dedicated hospitals opened, especially in adults but also in pediatrics, as happened in our center of Palidoro.

The reduction of visits in EDs is likely related to a combination of factors, such as a reduction in presentations of viral exposure due to social distancing, reduction in school-related possible stress and parents or carers deciding to stay at home due to fear of attending during a pandemic situation. In fact, respiratory tract infections, and other communicable diseases were the most frequent cause of ED visits before the pandemic (2019) and significantly decreased during the COVID-19 period (2020) (Table 1). In a French study, Angoulvant et al. [25] found a significant decrease over 70% of acute, gastro-enteritis, common cold, bronchiolitis and acute otitis media compared to the expected values, suggesting that restrictive measures had a critical impact on the transmission of infectious disease, more specifically on viral and viral-induced disease. The same considerations can be applied for the reduction of hospitalizations, less pronounced in our center of Palidoro being a regional COVID center (see Table 2 for detail). Despite the general reduction, the decrease of urgent hospitalizations was less sharp than ED visits (Fig. 2). The reduction of respiratory pathology admission such as asthma is related to changes in respiratory virus infections (ie reduction of the contribution of person-to-person transmission of respiratory viruses) and also to less exposure to inhalant allergens related to facial mask and lockdown [26, 27]. In addition to potentially changing levels of outdoor air pollutants, COVID-19 related public health interventions likely influenced pollution exposure profiles of children in other ways, including via decreased commuting and outdoor activity [27]. Another mechanism to reduce allergic responses include include the physical filtration of face masks and the potentially modified physiological response to allergens by breathing humid and hot air [28].

Nevertheless, as reported in a previous study performed in our hospital, we did not analyze the appropriateness of ED access; it is possible that the reduction of ED visits was partly due to a decrease of hospital use for conditions that could be safely managed at home or, in case of most vulnerable patients, by increasing use of telemedicine or remote telefonic consultations reserved to physician or parents [29].

In our study an elevation of pediatric emergency service by ambulance emerges in OPBG COVID center of Palidoro because the suspected patients were transported directly by the regional ambulance service and admitted directly to this center. Indeed, in our ED of Rome there has been a decrease in the number of ambulance arrivals possibly due also to more adequate use of emergency medical transport service, compared to the prepandemic period, in which “a substantial number of emergency medical transport service use is medically not indicated” as previously indicated by many authors [30–33].

Another important aspect emerged from our study is that fortunately the high/intermediate priority code consultation is less diminished in the two centers compared to the significative reduction of the low/non-urgent code (Table 1). While for non-urgent cases, hypothetically reduced ED use may have no public health consequences, a delay or failure to access patients with potentially serious and time-dependent diseases can lead to a worse prognosis for the patient, as a consequence of delay in prompt and proper diagnostic-therapeutic management. In order to reduce the possible diagnostic delay, the integration between territorial physician (family paediatrician in Italy) and hospital is strongly recommended. Parents of children affected by non-urgent diseases that can be managed by general practitioners have probably desisted from referring to the ED, following the government indications against crowding and being afraid of getting infected. Nevertheless, despite being an important contributor to pediatric ED overcrowding with the declaimed “best use of ED”, unnecessary visits are difficult to identify as to date recommend studies evaluating the appropriateness of ED use standardized, objective criteria tha clinician judgment alone with the development od evidence-based interventions to reduce non-urgent ED use [24, 34, 35].

In COVID-19 period during the lockdown in Italy, the possibility of postponing necessary urgent care for conditions such as acute appendicitis, diabetic ketoacidosis, abdominal or intracranial mass with possible serious consequences has been evocated in case reports [14, 15, 17, 19]. In our study, as proxy for probabile delayed access, we analyzed the rate of patients operated for complicated appendicitis (peritonitis) and we found no significant difference.

We have compared some pathologies (appendicitis, psychiatric diseases, hospitalization in the oncohematological environment) to verify eventual structured delays, but the comparison between COVID versus pre-COVID period has not shown significant results. Furthermore, we have not found an increase in serious diseases such as ketoacidotic diabetes, ileo-colic intussuscection and testis/ovary torsion. Furthermore, in all the considered pathologies we did not observe significant differences in the onset of symptoms between the 2 considered periods (prepandemic and pandemic). This could be explained by the changes implemented in our hospital during the COVID period, in addition to the differences between the pediatric way and the adult in terms of management and etiologies (ie stroke and acute cardiac disease in adults). Nevertheless, only a careful analysis over the next months will allow to verify if the particular attention to the warning signs / symptoms will have averted the diagnostic delay found in the first period of lockdowun by some authors [36].

In fact, we will carry out a further analysis of all pathologies possibly life-threatening or invalidating to verify which single pathology may have had a diagnostic delay and try to implement the counter-measuers to bring down the phenomenon of diagnostic delay. For this purpose, we have seen that in the category of tumors the numbers of diagnosis in ED has remained unmodified in the 2 considered periods, in agreement with an Ireland study [37]. Nevertheless, subsequent analysis must be considered for the reduction in disease such as hematological and neurological conditions, although in these cases some pathologies can be linked to infectious phenomena (Table 1), through further real-time longitudinal studies.

It is important to analyze the reasons for the large (− 64.6%) reduction in the number of subjects with headaches admitted to ED. In the previous years, data literature reported that the more frequent causes of non-traumatic headache in the ED include benign secondary headaches (35.4–63.2%), represented mainly of upper respiratory tract infections, sinusitis and other limited infections and primitive headaches (21.8–66.3%) [38]. The headache reduction of ED admission can be explained partly by respiratory tract infections, and other communicable diseases decrease, but a recent Italian multicenter survey showed that lifestyle modification represents the main factor impacting the course of primary headache disorders in children and adolescents. In particular, reduction in school-related stress with the reduction of school anxiety and school effort during the lockdown was the main factor explaining the significant improvement in the trend of headache, and intensity and frequency of attacks [39].

It is clear the reduced access of traumatic accidents is secondary to the almost complete restriction of outdoor, ludic and sport activity (and consequently of traffic) dictated by the government in this COVID-period. This is evitend in our study, albeit to a smaller extent than other diagnostic categories, especially if we consider the subjects who needed hospitalization, whereas most of them occurred within the home walls given the lockdown. The home quarantine during the lockdown may have been a risk factor for these events not only because children remained at home, but also because isolation increases the risk that vulnerable children experiencing neglect or maltreatment of domestic violence [29, 37]. In this unprecedented situation, pediatricians need to provide families with adequate education on home accident prevention, and to monitor the risk of domestic violence during all visits and telemedicine consultations [29]. Walker and Tolentino [13] reported an increase in the volume of calls to poison centers and the usefulness of an information campaign. The same Authors underline the importance of implementing strategies to prevent the lockdown from affecting the failure to resort to emergency care of patients with psychiatric pathology [13].

This study has several limitations. First, there is no data on the onset of symptoms. Second, there is no data on any contact with the family pediatrician prior to arrival in ED. Third, this is a hospital-based analysis that does not take into account the total pediatric access in the metropolitan area of ​​the city. However, we must note that the OPBG hospital as a pediatric hospital covers more than 50% of ED access under the age of 18 in the entire metropolitan area of Rome [40].

## Conclusions

During the COVID-19 pandemic, a reduction in the accesses in an ED of a tertiary children’s hospital was observed, mainly due to potentially deferrable conditions normally discharged from ED. This result demonstrates a great potential of the outpatient primary care system to reduce the use of the hospital for low risk conditions. Nevertheless, the decrease of urgent hospitalizations was less sharp than ED visits. The fear linked to the COVID-19 pandemic led to a possible “best use of ED” with major appropriateness of referrals. The correct application of the clinical pathways and the reorganization of the access to the ED could have the virtuous effect of a potential optimization of the available resources when the overcrowding reached alarming levels.

The data related to the reduction of urgent visits for respiratory tract infections and other communicable diseases during school closure and the national lockdown must make us reflect on the possible impact that these conditions (the most frequent cause of visits to ED before the pandemic) may have on the health system, in particular the ED, at the reopening of schools.

The major problem remains a fear for possible significant diagnostic delays in life-threatening or invalidating diseases. In this particular period, it is mandatory to encourage parents to interact with a general practitioner beftore turning to the ED, when necessary, to prevent the fear of COVID-19 from causing more harm than the virus itself, because serious diseases still occur.

Our study had not demonstrated a significant delay in some of the serious conditions such as tumors and peritonitis but we should keep in mind in agreement with Dann et al. [37] that, “due to their latency, these changes will take longer periods of time measurement to be definitively excluded”. Therefore, the challenge is to carry out more in depth studies to evaluate the health effect of the reduction of ED access for chronic and time-dependent conditions over short and long term period. A continuous and deep re-organizational process step by step of the ED is nececessary in the actually and upcoming COVID-19 pandemic situation.

A survey involving Italians pediatric scientific societies showed the need to give life a new approach for hospitalizations and outpatient visits through a greater use of telemedicine, educational programs on families and a more decisive role of family pediatricians is advocated to remodulate the pediatric health care, developing a program starting from the family and reaching the hospital through a new and efficient model of primary care [41].

In this particular period, it is mandatory to encourage parents to interact with a general practitioner or seen earlier to the ED, when necessary, to prevent the fear of COVID-19 from causing more harm than the virus itself, because serious diseases still occur. Finally, a deep re-organizational process of the ED was undertaken. As stated by Comelli et al. [42], a deadly revolution has begun: “We are living in it, we are living despite it”**.**

## Data Availability

Availability of data and material at Bambino Gesù Children Hospital.

## Abbreviations

COVID-19: Coronavirus Disease 2019
ED: Emergency Department
OPBG: Bambino Gesù Children’s Hospital (Ospedale Pediatrico Bambino Gesù)
HEIS: OPBG Healthcare Emergency Information System
HIS: OPBG Hospital Information System

## References

[1] Ghebreyesus TA.WHO Director-General’s opening remarks at the media briefing on COVID- 19 – 11 March 2020. 2020. Available at: https://www.who.int/dg/speeches/detail/who-director-general-s-opening-remarks-at-the-media-briefing-on-covid-19%2D%2D-11-march-2020. Accessed 22 Jul 2020.

[2] Protezione Civile. Chronology of main steps and legal acts taken by the Italian Government for the containment of the COVID-19 epidemiological emergency . Available from : http://www.protezionecivile.gov.it/documents/20182/1227694/Summary+of+measures+taken+against+the+spread+of+C-19/c16459ad-4e52-4e90-90f3-c6a2b30c17eb . Accessed 5 Aug 2020.

[3] Castagnoli R, Votto M, Licari A, et al. Severe acute respiratory syndrome coronavirus 2 (SARS- CoV-2) infection in children and adolescents: a systematic review. JAMA Pediatr. 2020;2. 10.1001/jamapediatrics.2020.1467. 10.1001/jamapediatrics.2020.1467 32320004

[4] The Novel Coronavirus Pneumonia Emergency Response Epidemiology Team (2020). Vital surveillances: the epidemiological characteristics of an outbreak of 2019 novel coronavirus diseases (COVID-19) — China, 2020. China CDC Weekly.

[5] COVID-19 National Incident Room Surveillance Team. COVID-19, Australia: Epidemiology Report 14 (Reporting week to 23:59 AEST 3 May 2020) [published correction appears in Commun Dis Intell (2018). 2020 May 15;44]. Commun Dis Intell (2018). 2020;44. 10.33321/cdi.2020.44.42.

[6] CDC COVID-19 Response Team (2020). Coronavirus disease 2019 in children – United States, February 12–April 2, 2020. MMWR Morb Mortal Wkly Rep.

[7] Centers for Disease Control and Prevention. Provisional COVID-19 death counts by sex, age, and state from the National Center for Health Statistics. Available at: https://data.cdc.gov/NCHS/Provisional-COVID-19-Death-Counts-by-Sex-Age-and-S/9bhg-hcku. Accessed 22 Jul 2020.

[8] Devulapalli CS. COVID-19 a mild disease in children. Tidsskr Nor Laegeforen. 2020;140(6). 10.4045/tidsskr.20.0231. 10.4045/tidsskr.20.0231 32321222

[9] Istituto Superiore di Sanità (2020). Sorveglianza Integrata COVID-19 in Italia.

[10] Critical preparedness, readiness and response actions for COVID-19 Interim guidance 24 June, 2020. https://www.who.int/publications/i/item/critical-preparedness-readiness-and-response-actions-for-covid-19. Accessed 22 Jul 2020.

[11] Bressan S, Buonsenso D, Farrugia R, Parri N, Oostenbrink R, Titomanlio L, et al. Preparedness and response to Pediatric CoVID-19 in European Emergency Departments: a survey of the REPEM and PERUKI networks [published online ahead of print, 2020 May 15]. Ann Emerg Med. 2020. 10.1016/j.annemergmed.2020.05.018. 10.1016/j.annemergmed.2020.05.018 PMC7225691 32419713

[12] Freund Y (2020). The challenge of emergency medicine facing the COVID-19 outbreak. Eur J Emerg Med.

[13] Walker DM, Tolentino VR (2020). COVID-19: the impact on pediatric emergency care. Pediatr Emerg Med Pract.

[14] Lazzerini M, Barbi E, Apicella A, Marchetti F, Cardinale F, Trobia G (2020). Delayed access or provision of care in Italy resulting from fear of COVID-19. Lancet Child Adolesc Heal.

[15] Ciacchini B, Tonioli F, Marciano C, Faticato MG, Borali E, Pini Prato A (2020). Reluctance to seek pediatric care during the COVID-19 pandemic and the risks of delayed diagnosis. Ital J Pediatr.

[16] Lynn RM, Avis JL, Lenton S, Amin-Chowdhury Z, Ladhani SN. Delayed access to care and late presentations in children during the COVID-19 pandemic: a snapshot survey of 4075 paediatricians in the UK and Ireland. Arch Dis Child. 2020;archdischild-2020-319848. 10.1136/archdischild-2020-319848. 10.1136/archdischild-2020-319848 32586927

[17] Snapiri O, Rosenberg Danziger C, Krause I, Kravarusic D, Yulevich A, Balla U (2020). Delayed diagnosis of paediatric appendicitis during the COVID-19 pandemic. Acta Paediatr.

[18] Health Policy team. Delayed access to care for children during COVID-19: our role as paediatricians - position statement. RCPCH 2020. https://www.rcpch.ac.uk/resources/delayed-presentation-during-covid-19-position. Accessed 14 Sept 2020.

[19] Wise J (2020). Covid-19: Delays in attending emergency departments may have contributed to deaths of nine children. BMJ.

[20] Lazio R (2020). Indicazioni operative per la gestione e la sorveglianza dei casi sospetti di infezione da nuovo Coronavirus (2019 - nCoV). Prot. n. 69913.

[21] Cozzi G, Zanchi C, Giangreco M, Rabach I, Calligaris L, Giorgi R, et al. The impact of the COVID-19 lockdown in Italy on a pediatric emergency setting. Acta Paediatr. 2020;29. 10.1111/apa.15454. 10.1111/apa.15454 PMC7361857 32598519

[22] Iozzi L, Brambilla I, Foiadelli T, Marseglia GL, Ciprandi G. Paediatric emergency department visits fell by more than 70% during the COVID-19 lockdown in Northern Italy [published online ahead of print, 2020 Jul 4]. Acta Paediatr. 2020. 10.1111/apa.15458. 10.1111/apa.15458 PMC7361573 32623774

[23] Pata D, Gatto A, Buonsenso D, Chiaretti A. A COVID-19 outbreak's lesson: Best use of the paediatric emergency department [published online ahead of print, 2020 Jun 2]. Acta Paediatr. 2020. 10.1111/apa.15386. 10.1111/apa.15386 PMC7300979 32488953

[24] O'Cathain A, Connell J, Long J, Coster J (2020). Clinically unnecessary’ use of emergency and urgent care: a realist review of patients’ decision making. Health Expect.

[25] Angoulvant F, Ouldali N, Yang DD, Filser M, Gajdos V, Rybak A, et al. COVID-19 pandemic: Impact caused by school closure and national lockdown on pediatric visits and admissions for viral and non-viral infections, a time series analysis. Clin Infect Dis. 2020:ciaa710. 10.1093/cid/ciaa710. 10.1093/cid/ciaa710 PMC7314162 33501967

[26] Kenyon CC, Hill DA, Henrickson SE, Bryant-Stephens TC, Zorc JJ (2020). Initial effects of the COVID-19 pandemic on pediatric asthma emergency department utilization. J Allergy Clin Immunol Pract.

[27] Taquechel K, Diwadkar AR, Sayed S, Dudley JW, Grundmeier RW, Kenyon CC (2020). Pediatric asthma health care utilization, viral testing, and air pollution changes during the COVID-19 pandemic. J Allergy Clin Immunol Pract.

[28] Dror AA, Eisenbach N, Marshak T, Layous E, Zigron A, Shivatzki S (2020). Reduction of allergic rhinitis symptoms with face mask usage during the COVID-19 pandemic. J Allergy Clin Immunol Pract.

[29] Ciofi Degli Atti ML, Campana A, Muda AO, Concato C, Ravà L, Ricotta L (2020). Facing SARS-CoV-2 pandemic at a COVID-19 regional children’s hospital in Italy. Pediatr Infect Dis J.

[30] Camasso-Richardson K, Wilde JA, Petrack EM (1997). Medically unnecessary pediatric ambulance transports: a medical taxi service?. Acad Emerg Med.

[31] Kost S, Arruda J (1999). Appropriateness of ambulance transportation to a suburban pediatric emergency department. Prehosp Emerg Care.

[32] Lowthian JA, Cameron PA, Stoelwinder JU, Curtis A, Currell A, Cooke MW (2011). Increasing utilisation of emergency ambulances. Aust Health Rev.

[33] Poryo M, Burger M, Wagenpfeil S, Ziegler B, Sauer H, Flotats-Bastardas M, Grundmann U, Zemlin M, Meyer S (2019). Assessment of inadequate use of pediatric emergency medical transport services: the pediatric emergency and ambulance critical evaluation (PEACE) study. Front Pediatr.

[34] Uscher-Pines L, Pines J, Kellermann A, Gillen E, Mehrotra A (2013). Emergency department visits for nonurgent conditions: systematic literature review. Am J Manag Care.

[35] Paul JE, Zhu KY, Meckler GD, Park DK, Doan Q. Assessing appropriateness of pediatric emergency department visits: is it even possible? [published online ahead of print, 2020 Feb 3]. CJEM. 2020:1–4. 10.1017/cem.2019.473. 10.1017/cem.2019.473 32009600

[36] Isba R, Edge R, Jenner R, Broughton E, Francis N, Butler J (2020). Where have all the children gone? Decreases in paediatric emergency department attendances at the start of the COVID-19 pandemic of 2020. Arch Dis Child.

[37] Dann L, Fitzsimons J, Gorman KM, Hourihane J, Okafor I (2020). Disappearing act: COVID-19 and paediatric emergency department attendances. Arch Dis Child.

[38] Raucci U, Della Vecchia N, Ossella C, Paolino MC, Villa MP, Reale A (2019). Management of Childhood Headache in the emergency department. Review of the Literature. Front Neurol.

[39] Papetti L, Loro PAD, Tarantino S, Grazzi L, Guidetti V, Parisi P (2020). I stay at home with headache. A survey to investigate how the lockdown for COVID-19 impacted on headache in Italian children. Cephalalgia..

[40] PREVALE (2020). Programma Regionale Valutazione degli Esiti degli Interventi sanitari.

[41] Lubrano R, Villani A, Berrettini S, Caione P, Chiara A, Costantino A (2020). Point of view of the Italians pediatric scientific societies about the pediatric care during the COVID-19 lockdown: what has changed and future prospects for restarting. Ital J Pediatr.

[42] Comelli I, Scioscioli F, Cervellin G (2020). Impact of the COVID-19 epidemic on census, organization and activity of a large urban Emergency Department. Acta Biomed.

